# Cultural tailoring for the promotion of Hepatitis B screening in Turkish Dutch: a protocol for a randomized controlled trial

**DOI:** 10.1186/1471-2458-10-674

**Published:** 2010-11-05

**Authors:** Ytje JJ van der Veen, Onno de Zwart, Johan Mackenbach, Jan Hendrik Richardus

**Affiliations:** 1 Erasmus MC, University Medical Center Rotterdam, Dept. of Public Health, the Netherlands; 2 Municipal Public Health Service GGD Rotterdam-Rijnmond, the Netherlands

## Abstract

**Background:**

Chronic hepatitis B virus infection (HBV) is an important health problem in the Turkish community in the Netherlands, and promotion of screening for HBV in this risk group is necessary. An individually tailored intervention and a culturally tailored intervention have been developed to promote screening in first generation 16-40 year old Turkish immigrants. This paper describes the design of the randomized controlled trial, which will be used to evaluate the effectiveness of the two tailored internet interventions as compared to generic online information on HBV, and to assess the added value of tailoring on socio-cultural factors.

**Methods/Design:**

A cluster randomized controlled trial design, in which we invite all Rotterdam registered inhabitants born in Turkey, aged 16-40 (n = 10,000), to visit the intervention website is used. A cluster includes all persons living at one house address. The clusters are randomly assigned to either group A, B or C. On the website, persons eligible for testing will be selected through a series of exclusion questions and will then continue in the randomly assigned intervention group. Group A will receive generic information on HBV. Group B will receive individually tailored information related to social-cognitive determinants of screening. Group C will receive culturally tailored information which, next to social-cognitive factors, addresses cultural factors related to screening. Subsequently, participants may obtain a laboratory form, with which they can be tested free of charge at local health centres. The main outcome of the study is the percentage of eligible persons tested for HBV through to participation in one of the three groups. Measurements of the outcome behaviour and its determinants will be at baseline and five weeks post-intervention.

**Discussion:**

This trial will provide information on the effectiveness of a culturally tailored internet intervention promoting HBV-screening in first generation Turkish immigrants in the Netherlands, aged 16-40. The results will contribute to the evidence base for culturally tailored (internet) interventions in ethnic minority populations. An effective intervention will lead to a reduction of the morbidity and mortality due to HBV in this population. This may not only benefit patients, but also help reduce health inequalities in western countries.

**Trial Registration:**

The Netherlands National Trial Register NTR 2394.

## Background

Hepatitis B virus infection (HBV) is one of the major infectious diseases in the world [1]. Each year, around 1,800 HBV infections, 79% of which are chronic, are reported in the Netherlands [2]. Chronic HBV infections cause 23% of all liver cancers in the Netherlands, and are an important problem in the Turkish community, which is the Netherlands' largest group of immigrants from non-industrialized countries [3,4]. While this community represents 8% of the total city population in Rotterdam (with 45,415 people), it accounts for 30% of reported chronic HBV infections [5]. Seventy percent of reported infections among Turks involve people aged between 16 and 40 years. In this age-category, the mean incidence of reported HBV infections is 122 per 100,000 individuals of Turkish origin, much higher than the 35 infections per 100,000 persons reported in the total population of Rotterdam [6]. However, these figures underestimate the population prevalence: many chronic HBV patients do not have the signs and symptoms of disease, and are therefore not reported. Population-based studies indicate a prevalence of chronic HBV of 0.2% in the general Dutch population, and a much higher prevalence of 2.6 - 4.8% in first generation Turkish immigrants (i.e. those born in Turkey) [4,7-9]. These studies furthermore show that the prevalence of chronic HBV in second generation Turks is similar to the general Dutch population.

The majority of Turkish patients with chronic infection have acquired HBV through infection from mother to child at birth, or through infection at a young age by blood contact with household members [10,11]. Carriers of the virus may infect others by blood contact or, later in life, through sexual contact [12].

In Turkey, every newborn is vaccinated in the first 24 hours since 1998 [13]. Furthermore, adolescent catch-up vaccinations ensured that adolescents up to the age of 16 years had been vaccinated by 2008[14]. Current national HBV-control policies in the Netherlands focus on screening pregnant women and on vaccinating specific risk groups, such as newborns from HBV-infected mothers (since 1989), children with at least one parent from an HBV-endemic area (since 2003), and people with high-risk behaviour (since 2002) [12]. These programmes leave a substantial part of the adult Turkish population in the Netherlands undetected and unprotected regarding HBV. Furthermore, in the past decade, treatment options of chronic HBV have improved [15]. In order to detect individuals eligible for treatment and to prevent transmission, screening for HBV should be promoted through public health interventions targeted specifically in first generation Turkish immigrants.

Tailoring interventions to determinants of health behaviour has proven to be effective in health promotion [16], also related to infectious diseases [17,18], and in promotion of screening participation [19,20]. Relevant social-cognitive determinants for this specific target population, derived from common health behaviour theories, focus group discussions, and a survey questionnaire in the Turkish population in the Netherlands [21,22] (YJJ van der Veen et al.: Social-cognitive and socio-cultural predictors of hepatitis B screening behaviour in Turkish Dutch, submitted), were the low awareness and knowledge regarding hepatitis B and its prevention, and the attitude, self-efficacy, social support and subjective norm regarding hepatitis B-screening in the target population. Up to now, tailored interventions are most often based on such individual factors, also called the proximal determinants of health behaviour. However, these proximal determinants may be dependent on more distal social-cultural factors [23]. These factors ask for 'cultural tailoring' of interventions including cultural traditions, values, and norms in tailored strategies [24,25]. In our previous work regarding the determinants of HBV-screening behaviour, we also identified socio-cultural factors related to HBV-screening. These were shame and stigma regarding HBV, the association of HBV-screening with sexuality, the importance of family values, religious values and rules regarding health, and the level of satisfaction with the Dutch health care.

A relatively easy way of tailoring health information for specific groups is using information and communication technology (ICT) and the Internet. Advantages for health promotion include the interactivity, use of active learning methods, multimedia presentation, temporal flexibility, and low costs relative to its potential population reach [26]. An important condition for success of tailored internet interventions is basic access to the internet. Research in the Netherlands has shown an increase in access to computers and the Internet in the population in general, and in the Turkish community as well [27]. In our survey, we found that 87% of the Turks had a computer at home, and that an equal part of the population used the internet. The majority (90%) of men and the younger women (16-28 years) who used the internet did this daily or at least a few times per week. Seventy-five percent of the women above the age of 28 years said to use the internet frequently. Of the 20% of women in this age group who said not to use internet themselves, about 70% reported to live with someone in the house who did make use of the internet.

The effect of tailoring in behaviour-focussed infectious disease control and the added value of including cultural tailored approaches has not been studied to date. We therefore recently developed two internet interventions aiming to promote screening in first generation Turkish immigrants aged 16 to 40 years, using the Intervention Mapping approach (YJJ van der Veen et al.: Development of a culturally tailored internet intervention promoting hepatitis B screening in the Turkish Dutch community, submitted). Intervention Mapping (IM) describes the stepwise process for the development of theory- and evidence based and practice-based interventions [28].

Tailoring is defined as any combination of information or change strategies intended to reach one specific person, based on characteristics that are unique to that person, related to the outcome of interest, and have been derived from an individual assessment [29]. The individually tailored intervention focuses on social-cognitive determinants of screening behaviour, such as awareness, knowledge, attitude, self-efficacy, perceived subjective norm and support, susceptibility to HBV, and personal norms related to health. Cultural tailoring may be defined as tailoring a health message 'which recognizes and reinforces a group's cultural values, beliefs, and behaviours and built upon those to provide context and meaning to the message about a given health problem or behaviour' [25]. Cultural tailoring is expected to have even more impact on behaviour than tailoring, by paying attention to the embeddedness of human health behaviour in the cultural context and social structure [30]. Cultural sensitivity in tailoring can be conceptualized in terms of two primary dimensions: surface structure and deep structure [25]. We used surface structure elements in order to increase the comprehension and acceptance of messages, by matching intervention materials and messages to characteristics of the target population, such as the language and role models preferred by the target audience. We used deep structure elements to convey salience, by understanding how members of the priority population perceive the cause, course, and treatment of hepatitis B, as well as how they perceive the determinants of the desired screening behaviour. We addressed factors such as religion and family values that influence screening behaviour. A detailed description of the intervention is described elsewhere (YJJ van der Veen: Development of a culturally tailored internet intervention promoting hepatitis B screening in the Turkish Dutch community, submitted).

This study aims to evaluate the effectiveness of the two tailored internet interventions as compared to generic online information on HBV, and to assess the added value of additional tailoring on socio-cultural factors.

## Methods/Design

### Study design

We apply a cluster randomised controlled trial to study the effect of the individually tailored internet intervention (group B) and the culturally tailored internet intervention (group C) on screening behaviour, compared to generic online information (group A). Measurements of screening behaviour (i.e. being screened for HBV (yes/no)) and of the determinants of this behaviour will be at baseline and one month post-intervention.

### Study population

The study population consists of 16-40 year-old citizens of Rotterdam, born in Turkey. Excluded from the study are those:

- not registered in the municipal population registers (MPR)

- aware of having been tested, and knowing to be a carrier of HBV

- aware of having been tested, and being immune

- aware of having been fully vaccinated

### Recruitment of the study population

From the municipal public registration (MPR) we will retrieve names and address details of all persons aged 16-40 years (as per 1 September 2010) who are first generation Turkish immigrants. All subjects will be provided with a unique client ID, which we have randomly assigned to one of the three intervention groups. Respondents will be recruited to visit the website by means of a personal invitation by postal mail. The invitations will be sent out from 13 September to 17 December, 2010. This period was chosen because of the end of the Ramadan on 9 September, 2010. The planning will ensure that all persons living at the same house address will receive the invitation on the same day. Simultaneously, an information campaign in the Turkish community in Rotterdam will be conducted, using newspapers, local radio, community-based organisations and key figures in the Turkish community. Additionally, respondents will be recruited through links on websites with general health information and websites directed specifically at the Turkish community.

In the personal invitation, the addressees will be referred to the internet. On the homepage of the project a short explanation of the health problem of HBV is given, together with information about the facilities provided at the website. Visitors of the website are only able to log in on the website by using their client ID provided in the personal invitation. The website will be offered bilingually, i.e. the visitor may choose between the Dutch or Turkish language. Visitors will then be guided through some exclusion questions in order to select persons eligible for testing (see exclusion criteria).

### Randomisation and exposure to the intervention

All persons living at the same address (i.e. family members or house-mates), will be assigned randomly to one of the three research groups: standard information (group A), individually tailored information (group B) and culturally tailored information (group C). Participants in the control (group A) and intervention groups (groups B and C) are thus enrolled in the same way. Those who enter the internet page and show to be eligible for testing will be questioned for demographic information and asked for a current email address for participation in a follow-up questionnaire. When participants stop during the internet session, and log in using their client ID later on, they will continue in the same group.

During the intervention, participants provide information on both social-cognitive and socio-cultural determinants by answering questions on their beliefs and expectations regarding hepatitis B screening. This information and the provided demographic data will be linked to the client ID and saved in a database. At the end of the information session or tailored intervention, participants may choose to receive a laboratory form either by email or post. Prior to receiving the form, they will receive client information, in which the procedure of testing, possible results of the test and follow-up is explained. Participants will then be asked to sign a checkbox for informed consent. Participants are also asked to co-operate in a follow-up questionnaire about the website one month after the date of first website visit. The laboratory form will be either sent by email or mail, and the client information is once more included. When the participant is younger than 18 years at the date of printing the form, a signed consent from the parents is needed on the form. In that case, information for the parents will be included in the client information. For individuals unable to use the internet, we will provide the laboratory form with generic information on HBV on request by postal mail. However, these individuals are excluded from the research population, as they are not exposed to one of the three interventions.

### Test site (location for blood sampling)

**Figure 1:**
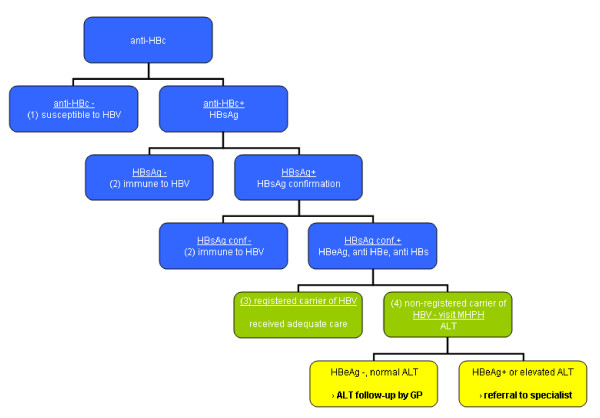
**Standard test algorithm for Hepatitis B screening in Turkish Dutch**.

Test sites (n = 85) are community health centres conveniently located in the neighbourhoods where participants live. The blood samples will be analysed according to a predefined standard test algorithm (see Figure 1). The laboratory will inform the Municipal Public Health Service (MPHS) about the test result by providing client ID, date of birth, postal code and test results.

### Follow-up actions

**Figure 2:**
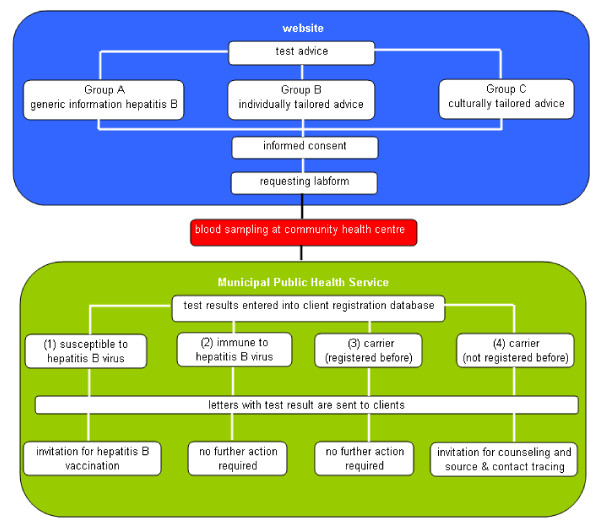
**Flowchart Hepatitis B screening in Turkish Dutch**.

All HBc-negative results (indicating that persons have not been in contact with the virus) can be sifted out by non-medical staff of the MPHS. All HBc-postive results will be assessed by a medical doctor. Accordingly, all test results will be entered into the client registration system of the MPHS based on name of the person (which may be retrieved using the client ID). This client registration system will automatically distribute a standard letter with the results to the participant. The four possible outcomes of the test are: (1) being susceptible to HBV; (2) being immune for HBV due to previous infection; (3) being a HBV carrier, already registered with the MPHS; (4) being a carrier, not registered with the MPHS yet (see Figure 1).Susceptible persons will be informed about their test result, and will be advised to be vaccinated (at a reduced rate) at the MPHS. Immune persons are informed about their test result and the fact that no further action is required. MPHS registered carriers will be informed about their result, and that no further action is required as they have received adequate care in the past. Non-registered carriers will be informed about their status, and will be requested to visit the MPHS for a counselling session and a second blood sample for determination of the liver function through an ALT test. Elevated ALT levels indicate liver inflammation, and these participants will be referred to a medical specialist. In case of normal ALT levels, the participants are referred to their GP for a yearly check-up. In all letters, a telephone number for additional questions is provided. Figure 2 represents the project's flow-chart, indicating the flow from the moment the participant logs in on the project website, through a visit to the community health centre for blood sampling, and the remaining procedure at the MPHS once the test result is known.

### Follow-up measurements

If test results have not been received by the MPHS four weeks after the laboratory form request, participants who have indicated to wish to be reminded of the testing, are sent a reminder email. Five weeks after the first log-in, participants who provided an email-address and have given consent for being approached for further research, will receive an email with a link to the website where a short questionnaire on social-cognitive and socio-cultural determinants, and the perceived quality of the intervention is presented. After completion of the questionnaire, participants may indicate whether they want to join in a raffle (based on email-addresses) of gift vouchers, as a token of appreciation for their participation.

### Sample size

The size of the total first generation Turkish population in Rotterdam aged 16-40 years is approximately 10,000. We expect that after having received the letter, approximately 60% of this population will go to the website (n = 6000, i.e. 2000 visitors in each group), and that half of that group will receive a test-advice and laboratory form (n = 3000, 1000 in each arm of the study). We hypothesize that in the three groups (standard information, individually tailored information and cultural tailored information) 20%, 35% and 45%, respectively, will go to the test location and be tested for HBV (n = 200, n = 350, n = 450, respectively). Accordingly, power calculations for the difference in compliance with the advice between the tailored and cultural tailored group, show a power of more than 0.90. Because of clustering in families (we randomize by house address), we take into account the effect of the cluster 'family', which may affect the power. Therefore we take a power of 0.90 as acceptable instead of the standard 0.80. Our hypothesis would lead to a total of 1000 tested persons, approximately 10% of the total target population. Based on responses in other studies, we expect that this predicts the actual response reasonably well [31-34].

### Measures

During the intervention, we will gather the following information:

Demographic factors: gender, age, socio-economic status (SES) of the residential area (categorized in low-mid/high SES by postal code), marital status, level of education (low-medium-high), income situation, type of health insurance, religion, and whether the person knows someone with HBV.

Social-cognitive factors: awareness, knowledge, attitude, self-efficacy, perceived subjective norm and support, susceptibility to HBV, personal norms related to health and the screening intention.

Socio-cultural factors: satisfaction with the Dutch health care and perceived rules regarding health and disease.

### Primary outcome measure

The primary outcome variable is having been tested due to the intervention (yes/no). The data will be available from the client registration system from the MPHS.

### Secondary outcome measures

The follow-up questionnaire will provide information on secondary outcomes by measuring the change in the following determinants one month after logging in on the website:

Awareness: In the past three months, did you think about a hepatitis B test? Answering options: never heard of a HBV-test, never thought of a HBV-test, heard of a HBV-test but not decided, heard of a HBV-test and decided (not) to take it, have had a HBV-test. The measure was based on the Precaution Adoption Process Model (PAPM) [35] and adapted from Costanza [36]. The awareness score may range from 0 to 5.

Knowledge is measured by five statements in order to provide tailored information about the most important issues of HBV for this population. The items were on the contagiousness of HBV, the main route of transmission in Turks, the occurrence in Turks, the serious consequences of HBV and the prevention of HBV. The knowledge score may range from 0-5.

Attitude is measured by six items about three pro's (each may score 1 point) and three con's (each may score -1) of HBV testing. The attitude score may range from -3 to 3.

Self-efficacy is measured by 4 items (on a scale from 1-10) about the ability to discuss testing with parents and/or partner, the ability to communicate with a doctor about the test, and to arrange testing.

Subjective norm is measured by asking whether the participant thinks parents and/or partner feels testing is important (scale 1-10).

Social support is measured by multiplying the scores on two questions: 1.) whether the participant thinks parents and/or partner will support the participant in testing (yes (1)/no (-1)), and 2.) whether this is important for the participant (yes (1)/no (0)). The score may range from -1 (negative support) to 1 (positive support).

Susceptibility to HBV is measured by asking the participant to indicate how susceptible he/she feels regarding HBV, and to ask how he rates his susceptibility related to other inhabitants of the Netherlands. Both items are measured on a 10 point scale (very low chance, very high chance).

Personal norm is measured by two items 'I should care well for my own health' and 'I am responsible for the health of others' on a five point scale (agree (1) - disagree (5)).

Perceived rules is measured by asking persons who indicated to be religious whether they perceive rules in their religious community about: how to deal with health, how to prevent disease, being responsible for one's own health/the health of others. Answers may be yes (1) or no (0), which may result in a rules-score of 0-4.

Satisfaction with Dutch health care is measured on a five-point scale by three statements on the cost of health care, the experience of doctors, and satisfaction with the Dutch health care in general.

Screening intention is measured on a five-point scale by asking whether the participant intends to be tested within three months (for sure (5) - surely not (1)).

### Analysis

Because the primary outcome variable is the percentage of eligible persons having been tested due to the intervention (yes/no), we will perform univariate and multivariate logistic regression analysis. Independent variables that will be included in the regression analysis are the demographic, social-cognitive and socio-cultural factors.

### Process evaluation

The follow-up questionnaire addresses issues of quality by questioning: the comprehensibility, the reliability, the relevance, and the applicability of the content [37]. We will be able to assess the perceived reach by the number of returned invitations; and the perceived dose by the number of visitors and the number of components viewed (obtained from the website statistics).

### Ethical approval

The Medical Ethical Review Board of Erasmus MC, University Medical Center Rotterdam, approved this study.

## Discussion

This trial will determine the effectiveness of a cultural tailored internet intervention promoting HBV-screening in first generation Turkish immigrants, aged 16-40 years, living in Rotterdam.

It is difficult to estimate the expected response rate because this study is the first in this population using a culturally tailored internet approach. In order to generate sufficient power to show the value of the individually tailored and the culturally tailored intervention, we will need at least 1000 participants, 10% of the total target population. Regular public health interventions such as PAP smear testing for cervical cancer, show response rates in the range of 49.8% to 67.7% in Turkish immigrants in the Netherlands, with invitations from general practitioners having a higher response compared to invitations from the MPHS [38]. In a community project in which individuals were invited to be tested for hepatitis B (by mail for a personal consultation at the community centre), the response among the Turkish population was 26% [31]. A recently introduced Chlamydia screening programme in 16-29 year-old inhabitants of three area's in the Netherlands also used a combination of a personal invitation by post and the option to request a test package through the internet. This resulted in a response of 16% in the general population [33]. An important factor in motivating the invited people to participate might be the endorsement of our project by community leaders. We therefore have asked for the advice of community leaders in the development phase of the project. We also plan to specifically address community leaders and community-based organisations during the information campaign in the Turkish community in Rotterdam, using community-based organisations and key figures in the Turkish community. However, it remains to be seen whether the members of the target community that are involved, sufficiently represent their community in order to facilitate implementation of the intervention. Furthermore, next to the primary outcome (having been tested or not) it is important to learn from possible changes in social-cognitive and socio-cultural determinants related to HBV-screening. It has recently been shown that follow-up questionnaires have high drop-out rates [39]. The raffle of gift vouchers may help motivate people to fill out the questionnaire.

In the past decade, health promotion interventions have increasingly used the internet for the delivery of health messages tailored to the needs of the individual, and these have proven to be effective in changing behaviour [40,41], but the need for addressing cultural factors in tailored programmes has been emphasized [16]. Although it is widely accepted that disease prevention efforts should consider cultural factors when addressing the needs of diverse populations, there is little evidence that doing so enhances effectiveness [42]. To our knowledge, randomised controlled trials have only been used to measure the effect of cultural tailored messages on cancer prevention behaviour [24]. The results of this study will contribute to the general evidence base for culturally tailored (internet) interventions in ethnic minority populations.

This study also responds to a recent call for migrant screening on viral hepatitis [43]. Currently, chronic HBV infections in migrants are less likely to be detected than those in other high-risk populations, such as men who have sex with men or injecting drug users, who in the Netherlands are targeted for screening and vaccination. Therefore, migrant screening may not only benefit patients and reduce the burden of illness and costs for the health care system of long-term complications due to chronic HBV infection, but also help to reduce health inequalities in western countries.

## Competing interests

The authors declare that they have no competing interests.

## Authors' contributions

OZ, JM and JHR conceived of the study, and helped in the coordination and supervision. YV developed the intervention and conducted the randomised controlled trial. All authors read and approved the final manuscript.

## Pre-publication history

The pre-publication history for this paper can be accessed here:

http://www.biomedcentral.com/1471-2458/10/674/prepub
